# Light-Induced Vitamin C Accumulation in Tomato Fruits is Independent of Carbohydrate Availability

**DOI:** 10.3390/plants8040086

**Published:** 2019-04-03

**Authors:** Nikolaos Ntagkas, Ernst Woltering, Sofoklis Bouras, Ric C. H. de Vos, J Anja Dieleman, Celine C. S. Nicole, Caroline Labrie, Leo F. M. Marcelis

**Affiliations:** 1 Horticulture and Product Physiology, Wageningen University and Research, Droevendaalsesteeg 1, 6709 PB Wageningen, The Netherlands; ernst.woltering@wur.nl (E.W.); bourassofoklis@gmail.com (S.B.); leo.marcelis@wur.nl (L.F.M.M.); 2 Food and Biobased Research, Wageningen University and Research, Bornse Weilanden 9, 6708 WG Wageningen, The Netherlands; 3 Business unit Bioscience, Wageningen University and Research, Droevendaalsesteeg 1, 6709 PB Wageningen, The Netherlands; ric.devos@wur.nl; 4 Business unit Greenhouse Horticulture, Wageningen University and Research, Violierenweg 1, 2665 MV Bleiswijk, The Netherlands; anja.dieleman@wur.nl (J.A.D.); caroline.labrie@wur.nl (C.L.); 5 Signify Research, High Tech Campus 7, 5656 AE Eindhoven, The Netherlands; celine.nicole@signify.com

**Keywords:** vitamin C, ascorbic acid, carbohydrates, irradiance, myo-inositol, galacturonate

## Abstract

L-ascorbate (ASC) is essential for human health. Therefore, there is interest in increasing the ASC content of crops like tomato. High irradiance induces accumulation of ASC in green tomato fruits. The D-mannose/L-galactose biosynthetic pathway accounts for the most ASC in plants. The myo-inositol and galacturonate pathways have been proposed to exist but never identified in plants. The D-mannose/L-galactose starts from D-glucose. In a series of experiments, we tested the hypothesis that ASC levels depend on soluble carbohydrate content when tomato fruits ripen under irradiances that stimulate ASC biosynthesis. We show that ASC levels considerably increased when fruits ripened under light, but carbohydrate levels did not show a parallel increase. When carbohydrate levels in fruits were altered by flower pruning, no effects on ASC levels were observed at harvest or after ripening under irradiances that induce ASC accumulation. Artificial feeding of trusses with sucrose increased carbohydrate levels, but did not affect the light-induced ASC levels. We conclude that light-induced accumulation of ASC is independent of the carbohydrate content in tomato fruits. In tomato fruit treated with light, the increase in ASC was preceded by a concomitant increase in myo-inositol.

## 1. Introduction

L-ascorbate (ASC; vitamin C) is a phytochemical known for its antioxidant properties and positive effect on nutritional iron availability. It is essential for a healthy human body [1]. Humans are unable to synthesize ASC [2,3,4]. Therefore, they rely on a fruit- and vegetable-rich diet for sufficient amounts of ASC. ASC bioavailability in the human body is higher when ASC originates from plant products compared to artificial sources [5,6,7].

Improving the ASC content of edible plant parts is an interesting option to contribute to public health. This requires thorough understanding of the biochemical and physiological processes involved in ASC regulation in plants. When plants are exposed to higher light intensities, their ASC contents increase [8,9,10,11]. Tomato appears very responsive to light in terms of ASC levels: ASC content of tomato fruits increased when the fruit trusses were exposed to higher irradiances during cultivation [12,13,14]. Detached green mature tomato fruits that ripened under light, achieved up to 5 times higher ASC levels in their pericarp than fruits that ripened in the dark [15,16]. This response has been related to a light-induced increase in photosynthetic activity [15,17] and a direct signaling effect of light on ASC biosynthesis-related genes [13]. In order to optimize the light environment for ASC accumulation, a better understanding of the underlying physiological network is essential.

There are several proposed pathways of ASC biosynthesis in plants. The vast majority of the ASC pool comes from the D-mannose/L-galactose (D-man/L-gal) biosynthetic pathway, with D-glucose as the initial substrate and mannose and galactose as the intermediate products [18]. In view of this substrate–product relationship, the general hypothesis that the content of soluble carbohydrates (glucose, fructose, and sucrose) regulates the content of ASC emerged [19,20,21,22]. Besides the D-man/L-gal pathway, the galacturonate pathway has been identified in ripening strawberry fruits; this pathway synthesizes ASC from galacturonate, which is a product of cell wall breakdown [23]. Even though the galacturonate biosynthetic pathway has been thoroughly described, to date there is limited evidence for its contribution to the ASC pool [19]. Two more pathways for ASC biosynthesis, namely the myo-inositol pathway and the gulose pathway, have also been proposed to exist in plants [24,25]. Genes encoding different steps in the latter pathway have been identified and transgenic plants overexpressing the gene encoding one enzyme of the myo-inositol pathway (myo-inositol oxygenase) achieved higher ASC levels [26]. There is yet limited evidence for a major contribution of these alternative pathways to the ASC pool [19]. Thus, in non-genetically modified plants the D-man/L-gal biosynthetic pathway is the main route determining the size of the ASC pool and its activity is potentially affected by soluble carbohydrate availability.

Apart from being a substrate for ASC biosynthesis, carbohydrates play a signaling role in a variety of processes including the development of antioxidant networks [27]. In tomato fruits and broccoli florets, carbohydrate feeding resulted in an increase in the expression rates of certain genes involved in ASC biosynthesis, such as GDP-D-mannose pyrophosphorylase (VTC1), GDP-D-mannose 3,5-epimerase (VTC2), and L-galactose-1-P phosphatase (L-GalLDH), as well as recycling and turnover genes, such as ascorbate peroxidase (APX), monodehydroascorbate reductase (MDHAR), dehydroascorbate reductase (DHAR), and glutathione reductase (GR) [20,22]. Therefore, soluble carbohydrates appear to regulate ASC levels by acting not only as a substrate but also as signaling cues for ASC biosynthetic, recycling and turnover genes.

The hypothesis that the pool of soluble carbohydrates regulates the ASC levels has been tested in a variety of different plant species and tissues, though with variable results. Artificial carbohydrate feeding did not increase the ASC content of photosynthesizing tissue in both pea seedlings [28] and in Arabidopsis leaves [9]. However, soluble carbohydrate feeding of harvested broccoli inflorescences delayed ASC depletion during storage [20]. Whether availability of carbohydrates limits ASC biosynthesis in tomato fruits remains to be elucidated.

Soluble carbohydrates are the substrate for ASC biosynthesis via the D-man/L-gal biosynthetic pathway. Higher light intensities stimulate the biosynthesis of ASC [15]. We hypothesize that this stimulation of ASC accumulation by light might at least be partially caused by increased production of soluble carbohydrates. The aim of this research is to investigate whether ASC accumulation in tomato fruits is limited by soluble carbohydrate availability. To test this hypothesis, a number of experiments were conducted in order to obtain different levels of soluble carbohydrates in tomato fruits: (1) exposing detached fruits to different light intensities, (2) cultivation of plants with different fruit loads, and (3) artificial feeding of detached fruits with carbohydrates. In these experiments, ASC contents were quantified and related to the carbohydrate contents.

## 2. Results

### 2.1. Detached Fruits in Light and Darkness

**Figure 1:**
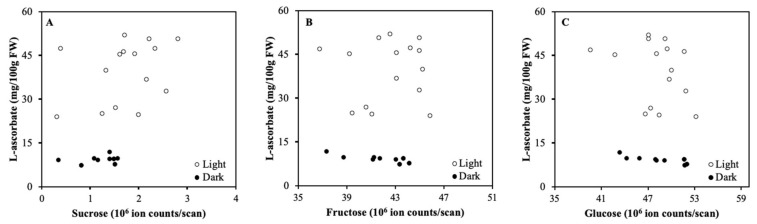
Correlation between L-ascorbate (ASC) and sucrose (**A**), fructose (**B**), and glucose (**C**) for detached tomato fruits that ripened in light (500 μmol m^−2^ s^−1^) or darkness for 15 days (Experiment 1). Each data point is based on pooled sample of pericarp discs of 2 fruits.

The aim of Experiment 1 was to investigate whether there is a correlation between carbohydrate content and ASC in detached fruits that matured under LED light or in darkness. In light, the ASC steadily increased during the experiment; in darkness ASC did not change [15]. Both in fruits stored in light and darkness the relation between the levels of ASC and individual carbohydrates (sucrose, fructose, and glucose) was not statistically significant (Figure 1A–C, respectively).

**Figure 2:**
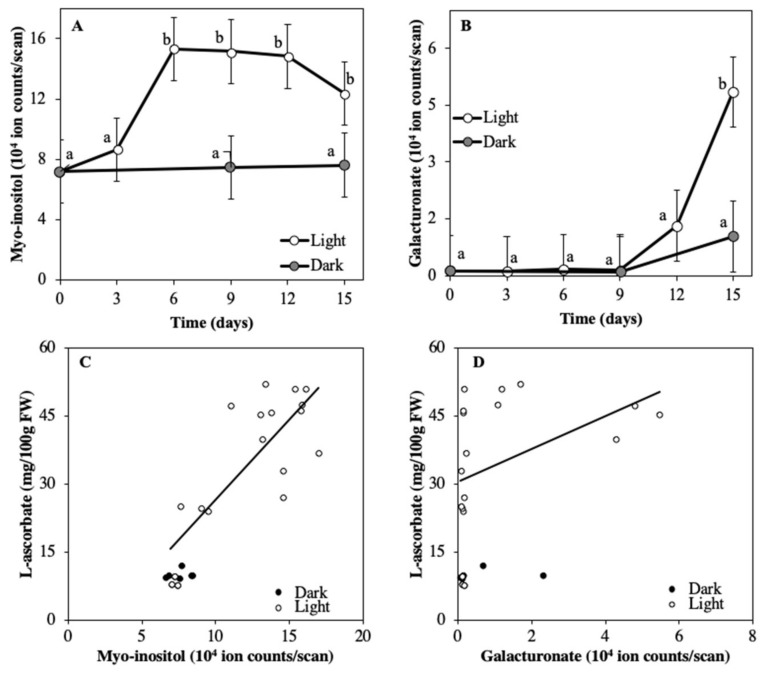
Relative level of ASC precursors myo-inositol (**A**) and galacturonate (**B**) in detached mature green tomato fruits stored in 500 μmol m^−2^ s^−1^ light (open symbols) and darkness (closed symbols) for 15 days. **C** and **D** show the relation between ASC and ASC precursors (myo-inositol and galacturonate, respectively) for the light and darkness treatments. Each data point is based on pooled sample of pericarp discs of 2 fruits. Solid lines in C and D represent a linear fitting on data points of the light treatment. Error bars represent standard errors of means, letters indicate statistical difference at α = 0.01; n = 5; Experiment 1).

The relative content of the ASC precursor myo-inositol in the fruits increased about 2-fold within six days of storage in the light (Figure 2A). From day 6 until the end of the experiment (day 15) myo-inositol remained stable. For fruits stored in darkness, no significant changes in myo-inositol were observed over time (Figure 2A). Galacturonate was relatively low in the fruits until day 9 in the light, thereafter its content increased rapidly (Figure 2B). ASC content in light showed a significant positive correlation with myo-inositol (r = 0.59, Figure 2C). The linear fitting however between ASC and myo-inositol was not significant for the dark treatment (r = 0.48). ASC significantly correlated with galacturonate for both the light (r =0.63) and dark (r = 0.98) treatments.

ASC levels of tomato fruit stored for 15 days in light and darkness were measured every three days. ASC in the light treatment increased approximately five-fold until day 9 when fruit entered the breaker stage (from 8 to 48 mg/100 g FW), and remained constant from day 9 until day 15. In the dark treatment, ASC did not show any significant changes from the beginning of the experiment until day 15 (8–11 mg/100g FW) [15].

### 2.2. Soluble Carbohydrate Content Varied by Fruits Load

**Figure 3:**
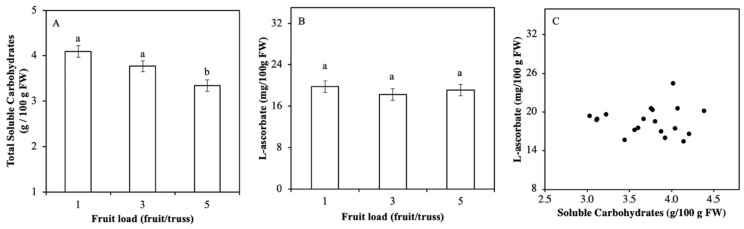
Relation between fruit carbohydrate and ASC levels in mature red tomato fruits grown at 3 different fruit load levels (1, 3, or 5 fruits per truss). Total soluble carbohydrate content (sum of sucrose, fructose, and glucose) (**A**) and ASC content (**B**). Correlation between ASC and carbohydrate content of individual samples (**C**). Each data point is based on pooled sample of pericarp discs of 2 fruits. Error bars represent standard errors of means, letters indicate statistical difference at α = 0.01; n = 5; Experiment 2).

The aim of Experiment 2 was to investigate whether carbohydrate content in red fruits, as varied by different fruit load levels, correlates with ASC content of tomato fruits that ripened on the vine. Reducing the number of fruits per truss resulted in significantly higher total carbohydrate levels when fruits had reached the red mature stage (Figure 3A). There were no significant effects of fruit load treatments on ASC content (Figure 3B). Hence, ASC did not correlate with carbohydrate content (Figure 3C).

**Figure 4:**
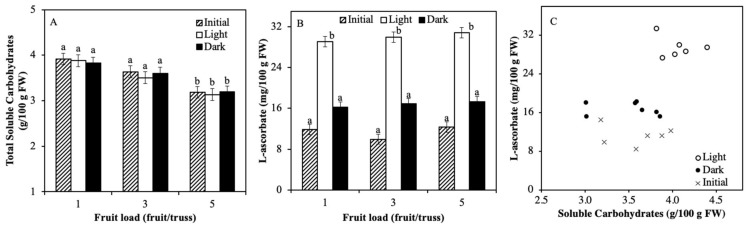
Relation between fruit carbohydrate and ASC levels in mature green and light or dark ripened tomato fruits that were grown at 3 different fruit load levels (1, 3, or 5 fruits per truss). Total soluble carbohydrate content (sum of sucrose, fructose, and glucose (**A**) and ASC levels (**B**)) before and after ripening in light 300 μmol m^−2^ s^−1^ or darkness for 7 days. Correlation between ASC and carbohydrate content of individual samples (**C**). Each data point is based on pooled sample of pericarp discs of 2 fruits. Error bars represent standard errors of means, letters indicate statistical difference at α = 0.01; n = 5; Experiment 3).

The aim of Experiment 3 was to investigate whether variable carbohydrate content in mature green fruits, as induced by different fruit load treatments, correlates with ASC in tomato fruits that were ripened under light (until red mature stage). Total soluble carbohydrate level in fruits increased significantly with lower fruit load but was not affected by light conditions during storage (Figure 4A). There were no significant differences in initial fruit ASC level among the three fruit load treatments (Figure 4B). Subsequent storage in light resulted in a significant increase of ASC of about 300% as compared to the initial content. Storage in darkness also resulted in an increase in fruit ASC but to a lesser extend (~70%) than storage in light. There was no significant effect of fruit load on ASC level of stored fruits at the end of the light treatment (Figure 4B). Linear regression analysis showed that there was no correlation between ASC and soluble carbohydrates both for the initial time point as well as at the end of the light and darkness treatments (Figure 4C).

### 2.3. Soluble Carbohydrate Content Varied by Truss Feeding

**Figure 5:**
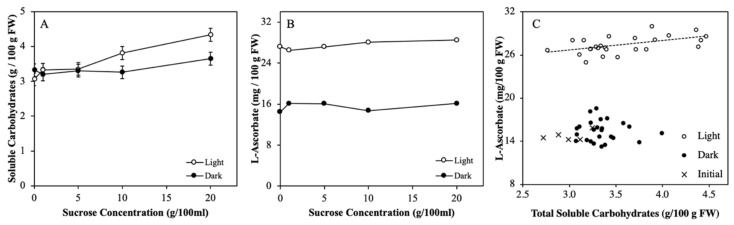
Effect of sucrose feeding on fruit total soluble carbohydrate content (sum of sucrose, fructose, and glucose (**A**) and ASC content (**B**)) in tomato when detached immature green fruits were fed with 5 different sucrose concentrations during 7 days of ripening until red in light and darkness. Correlation between ASC and soluble carbohydrates content for fruits in light and darkness (**C**). Line in C represents a linear fitting of data points of the light treatment. Each data point is based on pooled sample of pericarp discs of 2 fruits. Error bars represent standard errors of means, α=0.01; n=5; Experiment 4). Error bars in Figure B are smaller than symbol size.

The aim of Experiment 4 was to investigate whether different carbohydrate content as induced by sucrose feeding of the truss, correlates with ASC content in tomato fruits that ripened in light during the sucrose feeding. Tomato trusses were fed with sucrose solutions of different concentrations under light and dark conditions for seven days. Soluble carbohydrates of fruits fed with 20 % (*w*/*v*) sucrose solution and kept in light, were significantly higher than their initial content (data not shown) and the lower sucrose feeding levels at either light or darkness (Figure 5A). ASC content was significantly higher at the light treatment compared to the dark treatment. However, sucrose feeding did not affect the ASC content of the fruits in the light nor in darkness (Figure 5B). A linear regression showed only a significant correlation between ASC and soluble sugar content in the light (r = 0.53; Figure 5C).

Sucrose feeding did not result in higher respiration rates of the fruits: the average CO_2_ release rate was 18 mL kg^−1^ h^−1^ (± 0.2) for both the light and dark treatments. There were no significant differences in the rate of photosynthesis of fruits from different feeding treatments, with an average ΦPSII of 0.39 for both treatments. Fv/Fm was lower in the light compared to darkness; averaged over all sucrose feeding treatments Fv/Fm was 0.55 and 0.78 for light and darkness, respectively. This difference in Fv/Fm was most likely related to a slightly accelerated development of the fruit in light.

## 3. Discussion

### 3.1. Light Regulation of ASC is not via Soluble Carbohydrates

The D-man/L-gal ASC biosynthetic pathway is considered to contribute the most to the ASC pool in plants, unless genetically modified [19]. The D-man/L-gal pathway utilizes glucose in producing ASC in eleven steps with intermediate products such as D-mannose and L-galactose [18]. As glucose is the product of sucrose breakdown and the precursor of ASC in the D-man/L-gal pathway, we tested the hypothesis that soluble carbohydrate availability correlates with ASC content. High light intensity positively affects the ASC levels in treated mature green tomato fruits [15,16]. Here we showed in detached tomato fruits that when ASC accumulation was induced by light, there was no significant parallel change in soluble carbohydrates. When soluble carbohydrate content was varied, either by different fruit loads on the plant or by feeding sucrose to detached trusses, ASC levels did not change in parallel to the carbohydrate content. That was also the case for fruits with different carbohydrate content that were ripened under irradiance levels that stimulate the ASC biosynthesis. Since no correlation between the levels of soluble carbohydrates and ASC was found, carbohydrates are apparently not the causal the causal factor for differences in ASC levels in harvested fruit and are not determinate for the accumulation of ASC under light.

In the current work, we show the absence of a correlation between carbohydrates and ASC levels. This is in line with research in some other species and tissues. Exogenous application of sucrose or glucose in barley [21] and pea [28] embryonic axes did not cause ASC accumulation. ASC content of broccoli typically decreases with storage. Feeding of detached broccoli inflorescences did not result in ASC increase but in delayed ASC depletion [20]. In the same study, it was also found that sucrose feeding increased the expression rates of turnover (APX), recycling (MDHAR, DHAR, and GR), and biosynthetic (L-galactono-1,4-lactone dehydrogenase: GLDH) genes suggesting that carbohydrates regulate ASC through direct signaling. These signaling effects require further research using a variety of species as it has been suggested that carbohydrate regulation of ASC is species specific [29]. Feeding Arabidopsis plants with sucrose reduced ASC in the leaves due to inhibitory effects on ΦPSII [9]. This may suggest a regulatory role of photosynthesis for ASC in green tissue. The rate of photosynthetic electron transfer regulates the redox state of plastoquinone and that affects the ASC content through signaling on ASC enzymes [17]. In conclusion, it is most likely that the beneficial effect of light on ASC is mediated through photosynthesis [15] and signaling effects on expression of genes of the D-man/L-gal pathway [13], rather than by an increase in the substrate of ASC. Our research does not disprove that carbohydrate regulation of ASC in tomato could be induced after carbohydrate levels exceed a certain threshold. However, if there would be such a threshold, the commonly found carbohydrate concentrations in tomato fruits are above this threshold.

Incubation of micro-tom fruits in 5 g/100mL sucrose solution [22] led to an increase in ASC. This increase has been suggested to be due to a possible rise of the respiration rates due to carbohydrate feeding. The rate of respiration regulates the ASC content by regulation of the enzymatic activity of the last step (GLDH) of the D-man/L-gal biosynthetic pathway [30,31]. The effect of carbohydrate feeding on respiration rates differs between our research (no changes in respiration rates) and the previously mentioned case, potentially due to differences in plant material and feeding methods in between the two cases. This puts forward the idea that carbohydrates might affect the ASC levels only in the cases they regulate the rate of respiration but most likely not through direct signaling.

### 3.2. ASC Accumulation Coincides with Increased Availability of Myo-inositol and Galacturonate

Besides the D-man/L-gal pathway, other biosynthetic pathways for ASC have been proposed for plants. The galacturonate pathway produces ASC from the D-mannose released as a product of cell wall breakdown [23]. As D-mannose is an intermediate product of the D-man/L-gal pathway, ASC production through the galacturonate pathway might also be dependent on the D-mal/L-gal pathway and consequently on soluble carbohydrate availability. When mature green fruits were stored in light, an increase in ASC was observed. Galacturonate—a precursor in the secondary biosynthetic pathway—started to increase only after ASC had reached its highest level. This late increase of galacturonate (from six days after turning red) was most likely due to the progress of development (ripening) of the fruits and was not the consequence of increased activity of the D-mal/L-gal pathway as there was not an increase of carbohydrates. The rate of conversion of exogenously fed galacturonate to ASC is higher in mature red tomato fruits and the pathway is in vivo induced by ripening [22]. Even though the galacturonate pathway has been proposed to be a possible control point for ASC [32], there is to date no evidence that this pathway may contribute considerably to the ASC pool in non-genetically modified plants.

A four-step biosynthetic pathway that synthesizes ASC from myo-inositol with an intermediate precursor—L-gulono-1,4-lactone—has been proposed but not identified yet in plants [25,33]. The light-induced increase of ASC in mature green fruits that ripened under light was preceded by an increase in myo-inositol by three days. The fact that the substrate for ASC biosynthesis through the myo-inositol pathway was being built up ahead of the ASC response puts into perspective the potential contribution of this pathway to the ASC pool when tomato fruit are stored in the light. So far, ASC biosynthesis of considerable amounts of ASC has been observed only in overexpressors of steps of the myo-inositol pathway [26,34,35]. Overexpression of strawberry myo-inositol oxygenase (MIOX) in transgenic tomato plants lead to higher ASC content in the leaves only when myo-inositol is artificially fed [36]. This suggests that myo-inositol content is likely insufficient for ASC biosynthesis in leaves. However, as seen in Experiment 1, myo-inositol content might not be a restriction for ASC accumulation in tomato fruits. The myo-inositol pathway is probably unlikely in non-genetically modified plants [37]. Current research does not provide sufficient evidence for the contribution of the myo-inositol pathway in plants. However, as the levels of the precursor correlate with ASC levels in tomato, it would be worth investigating if the activity of this pathway in tomato fruit would be affected by myo-inositol feeding (e.g., radioactive isotopes).

## 4. Materials and Methods

### 4.1. Experiment 1—Detached Mature Green Fruits Stored in Light and Darkness

In Experiment 1 mature green tomato fruits (*Solanum lycopersicum* cv. Vimoso) were harvested in spring from a commercial glasshouse (Royal Pride Holland) in Middenmeer, the Netherlands. The fruits were taken from the 3rd and 4th positions of the truss (counting acropetally, starting from the oldest fruit present on the truss) from trusses of totally 8 fruits. After harvest, uniform fruits were selected according to color (−0.6 ± 0.03 NAI and 0.07 ± 0.02 NDVI color indices), size (6.2 ± 0.3 cm) and weight (43 ± 3 g). Light treatments took place in a climate-controlled room (Wageningen University and Research facilities) which contained 2 compartments: one with a light treatment (500 μmol m^−2^ s^−1^ of white light at fruit level and blue phosphorous-coated LEDs (GreenPower LED, Philips, The Netherlands) and the other in darkness. The LEDs were placed at a height of 80 cm above the fruits. All sides of the compartments were covered with neutrally reflective MC-PET sheets (SRF-A032T, Sekisui Plastics Co., LTD, Osaka, Japan) in order to improve light distribution. Irradiance difference between the brightest and darkest points of the area where the fruits were placed was 10 μmol m^−2^ s^−1^. In order to expose fruits to light uniformly, the fruits were placed in all spots of the illuminated area by daily rotation. The spectrum of the LED light treatment was measured with a spectroradiometer (USB2000, Ocean Optics, Duiven, The Netherlands; calibrated against a standard light source) and had a phytochrome stationary state [38] of 0.83. Air temperature was 18°C in all compartments. The temperature of the epidermis of the fruits in the light treatment was approximately 0.1°C higher than air temperature and the difference between the light and darkness treatments was less than 0.4°C. Air and fruit temperature was monitored with k-type thermocouples (shielded from direct radiation) on TC-08 data loggers (Picotechnology LTD., Cambridge, UK) calibrated in distilled water at freezing and boiling point. Relative humidity in the room was 70%. After removal of the calyx, the fruits were placed with the scar downwards in order to minimize fruit water loss. In these conditions the fruits in both treatments had lost 2.4% of their initial weight after 15 days. ASC was analyzed in fruit pericarp as in tomatoes the vast majority of ASC is found in the pericarp [22,39]. ASC was analyzed every 3 days until the end of the 15-day light and darkness treatments. The duration of this experiment was 15 days as ASC levels were observed to increase from day 0 to day 9 and then remained unchanged after the fruit entered the breaker stage (day 9 to day 15). The initially green-mature fruit reached the red-mature stage by the end of the 15 day treatment. From each treatment 10 fruits were paired into 5 replicates. Each replicate consisted of 6 pericarp discs (2 fruits, 3 discs per fruits, 1 cm diameter).

### 4.2. Experiment 2—Soluble Carbohydrate Content varied by Fruits Load

In Experiment 2 tomato seeds (*Solanum lycopersicum* cv. Komeett) were sown on August 6, 2015, shoots were grafted on a Maxifort rootstock and then topped, resulting in two stems per plant. On October 8 they were planted on rockwool slabs at a density of 2.5 stems per m^2^ in a glasshouse compartment at the research facilities of Wageningen University and Research, Bleiswijk. Average air temperature was 20 °C, ambient CO_2_ concentration was 600–800 ppm during daytime and relative humidity was 80%. In addition to the daily outside global radiation, 185 μmol m^−2^ s^−1^ of supplementary red and blue LED (95% red and 5% blue, Philips Greenpower) light was applied from midnight until sunset. Nutrient supply and pest and disease control followed commercial practices. Flowers were pollinated by bumble bees. In order to produce fruits with different carbohydrate content, 3 fruit load treatments were applied: trusses were pruned to either 1, 3, or 5 flowers per truss. This pruning was applied to all trusses of an individual plant. Fruits were harvested in March, at the red mature stage. Ten fruits per pruning treatment were paired into 5 replicates with each replicate consisting of 6 pericarp discs from two fruits. All fruits were harvested at the same time point. Each replicate originated from a different plant from a truss at the same height on the plant. The fruit load treatments were randomized over all 30 plants of the line.

### 4.3. Experiment 3—Detached Fruit with Variable Soluble Carbohydrate Content Due to Fruit Load, Are Exposed to Light and Darkness

In Experiment 3 tomato plants (*Solanum lycopersicum* cv. Komeett) were grown in a glasshouse compartment next to the compartment of Experiment 2. All conditions and fruit load treatments were similar as in Experiment2 unless otherwise stated. Sowing and transplanting dates were identical to Experiment 2. In this glasshouse compartment, far-red LEDs (research module far-red, Philips Greenpower) were additionally installed with an intensity of 40 μmol m^-2^ s^-1^. Fruits from different fruit load treatments were harvested at the mature green stage and stored for 7 days under darkness or at 300 μmol m^-2^ s^-1^ of white light (blue phosphorous-coated LEDs; GreenPower LED, Philips, The Netherlands) until ripening (red mature stage). Storage conditions were identical to Experiment 1. For the ASC and carbohydrate measurements, 10 fruits were paired into 5 replicates, and each replicate consisted of 6 pericarps from two fruits. Fruit load treatments were randomized over all 30 plants of the line.

### 4.4. Experiment 4—Truss Feeding Experiment

In Experiment 4 tomato trusses (*Solanum lycopersicum* cv. Vimoso) were taken from the same glasshouse as those of Experiment 1. The trusses were harvested when fruits were at the immature green stage (slightly smaller than mature green), in order to facilitate the sucrose uptake from the feeding. Intact trusses bearing 8 fruits per truss were harvested early in the morning. The cut stem end of the truss was submerged in tap water during transportation from the greenhouse to the lab in order to prevent air embolism of the xylem. Upon arrival at the lab of Wageningen University and Research, where the trial took place, 1 cm from the cut end of the truss stem was excised under water in order to remove possible cavitation that might have taken place during transportation. On the freshly cut end, 5 cm flexible silicon tube was attached. The tube was connected to falcon tubes (50 mL) containing sucrose solutions of different concentrations (20, 10, 5, 1, and 0 g/100mL H_2_O). To achieve the same osmotic potential in all solutions 0, 1.77, 2.52, 3.03, and 5.32 g of mannitol was added respectively to the 20, 10, 5, 1, and 0 g/100mL sucrose solutions. The cut end was recut by 0.5 cm every three days to remove any wound induced blockages. The tomato trusses attached to the feeding system were stored for 10 days in temperature-controlled cabinets. Irradiance was 280 μmol m^−2^ s^−1^ applied continuously, using the same white LEDs as in Experiment 1. This irradiance level should be sufficient to increase the ASC content considerably [15]. Temperature and relative humidity were identical to Experiment 1. After 10 days of sugar feeding the fruits had reached the red mature stage. At that time, fruits in positions 1 and 2 (counting acropetally, starting from the oldest fruit present on the truss) were analyzed for ASC. Ten fruits were paired into 5 replicates, each replicate consisting of half a tomato coming from two fruits.

### 4.5. ASA and Total Soluble Carbohydrates Determination

In Experiment 1, contents of ASC, soluble carbohydrates (sucrose, glucose, and fructose) and the ASC precursors myo-inositol and galacturonic acid were measured in the pericarp, using gas chromatography coupled to mass spectrometry (GC-MS) of derivatized polar compounds [40]. Methanol (0.7 mL) containing ribitol as an internal standard was added to 100 mg of frozen fruit powder, vortexed for 10 s, and then mixed for 10 min at 950 r.p.m. After 15 min sonication and 10 min centrifugation, 250 μL of the supernatant was re-extracted with 185.5 μL of chloroform and 375 μL of ultrapure water. Aliquots (50 μL) of the upper (polar) phase were dried in a speedvac overnight. Prior to GC-MS analysis, these dried extracts were derivatized online using a CombiPAL autosampler (CTC Analytics AG, Zwingen, Switzerland) and both methoxyamine and N-methyl-N-(trimethylsilyl) trifluoroacetamide (MSTFA) as derivatization agents. An alkane mixture (C10–C30) was automatically added to each sample. The derivatized polar extracts were then analyzed by a GC-MS system comprising an Agilent 6890 gas chromatographer (Agilent Technologies, Santa Clara, USA) coupled to a Pegasus III time-of-flight MS (Leco Instruments, Saint Joseph, USA). Target compounds were annotated by matching both the observed electron impact (EI) mass spectra and retention indexes with the Golm EI-spectral database (http://gmd.mpimp-golm.mpg.de/). Their relative levels were calculated from the corresponding GC-MS peak heights with correction for the internal standard.

In Experiments 2, 3, and 4, ASC and soluble carbohydrates were measured. The pericarp samples were frozen in liquid nitrogen immediately after dissection and mechanically grinded to fine powder under liquid nitrogen. Approximately 0.2 g of the powder was mixed with 0.5 mL 3.3% metaphosphoric acid and thawed on ice in darkness. After 10-min sonication (Branson 2200; Branson Equipment Co., Shelton, CT, USA), samples were centrifuged at 25,000 rcf at 4°C for 10 min. The supernatant was used for L-ASC determination. In 100 μL of each of these extracts L-DHA was reduced to ASC in a separate tube by the addition of 50 μL DTT 5mM and 400mM Tris base [41]. In this second extract, total ASC was determined allowing the calculation of DHA by subtracting the amount of reduced ASC. However, DHA was below the detection threshold and therefore not considered in our further analysis. Extracts were measured in a high-performance liquid chromatography system (ICS-5000, Dionex Corporation, Sunnyvale, USA). From the initial pulverized tomato sample, 0.3 g was taken for the determination of soluble carbohydrates. After 5 mL of 85 % ethanol was added, the samples thawed on ice and consecutively placed in thermal bath at 80 °C for 20 min and centrifuged at 8500 rpm at 4 °C for 5 min. The supernatants were transferred to Eppendorf tubes and dried in a SpeedVac (SPD 2010; Thermo Fisher Scientific Inc., Asheville, NY, USA) for 2 h. Carbohydrates were resuspended in 1 mL Milli-Q water and samples were sonicated for 10 min. After diluting the extract 10 times, soluble carbohydrates were quantified using high-pressure anion exchange chromatography (HPAEC; ICS5000; Dionex, Sunnyvale, CA, USA) by the use of an anion exchange column (250x-2 mm; CarboPac PA1; Dionex) at 25 °C. Chromatograms were analyzed in Chromeleon 7.0 software (Dionex) and glucose, fructose and sucrose were quantified using calibration curves of authentic standards.

### 4.6. Measurements of Respiration and Photosynthesis

The photosynthetic electron transport rate in photosystem II (ΦPSII) of the tomato fruits was measured under 300 μmol m^−2^ s^−1^ with a chlorophyll fluorescence imaging system (FluorCam 700MF, Photon System Instruments, Brno, Czech Republic). With the same system maximum quantum efficiency of photosystem II (Fv/Fm) after 30 min dark adaptation was also measured. Fv/Fm was measured under a saturating pulse of 3500 μmol m^−2^ s^−1^. FluorCam v.5.0 software was used to operate the measurement protocol in FluorCam 700MF. Ten replications (individual fruits) were done per treatment.

Fruit respiration was assessed by measuring CO_2_ exchange of the fruits. A portable infrared gas exchange system (LI-6400; Li-Cor Inc., Lincoln, Nebraska, USA) was used. A single tomato was placed in a transparent, plastic sphere, which was bypassed in the sampling circuit of the LI-6400. The leaf cuvette of the device was isolated from the sampling circuit [42]. The sphere reduced irradiance by 10% with no effects on the spectrum. Temperature in the sphere was 18,5 °C and CO_2_ concentration of the reference was set to 450ppm. The CO_2_ emission rate was logged for 15 min after a stabilization period of 15 min. Ten replications (individual fruit) were done per treatment.

### 4.7. Statistical Analysis

Two-way analysis of variance (ANOVA) was used to test the effects of two factors on ASC, ASC precursors, carbohydrates, respiration, and photosynthesis data: light treatment and time on L-ASC in Experiment 1, fruit load and light treatment in Experiment 3, and sucrose feeding and light in Experiment 4. One-way ANOVA was used to test the effects of fruit load on L-ASC (Experiment 2). In all experiments, the significance of carbohydrate content explaining variability in ASC was tested by linear regression analysis. The pool of material from two fruits was considered an independent replicate. This may have underestimated random variance. Therefore, the tests have been conducted at α = 0.01 instead of the commonly used α = 0.05 with post hoc Tukey’s honestly significant difference (HSD) multiple comparison tests (α = 0.01). Statistical analyses were carried out with GenStat 18th edition (VSN, International, Hempstead, UK).

## 5. Conclusions

Soluble carbohydrates are the precursor for ASC biosynthesis. Mature green tomato fruits when allowed to ripen under higher light levels achieve higher ASC content. As light may stimulate photosynthesis and consequently availability of sugars in the plant, we hypothesized that the positive effects of light on ASC content could be due to an increase in soluble carbohydrates. In a series of experiments, it has been shown that mature green tomato fruits achieve higher content of ASC when kept in light, but no correlation with the soluble carbohydrate content has been observed. When carbohydrate content was altered either by fruit pruning treatments or artificial truss feeding, no effects on ASC content was observed either. It is concluded that ASC accumulation by light is not due to an increase in soluble carbohydrates and that soluble carbohydrates do not limit ASC biosynthesis as increasing the carbohydrate concentration does not result in ASC increase. The increase of ASC in fruit stored in light was preceded by an increase in myo-inositol. However, further research is essential to determine if the myo-inositol biosynthetic pathway produces considerable amounts of ASC.
